# Right time, right place? New evidence on effective health workforce distribution and retention

**DOI:** 10.1186/1472-6963-14-S2-O19

**Published:** 2014-07-07

**Authors:** James Buchan

**Affiliations:** 1 University of Technology, Sydney, Australia & Queen Margaret University, Edinburgh, UK

## 

There is growing recognition that the achievement of health systems objectives, including UHC, requires an effective health workforce. This includes dimensions of effectiveness related to geographic distribution that allows access to services, and effective retention of scarce and often expensive skills within a sustainable workforce. (The latter issue is the counterpoint to health worker migration and mobility, which will be covered in another session at the conference).

There have been many policies aimed at improving health workforce distribution and retention, across all countries. Despite this growing policy interest, the evidence base remains fragmented.

This presentation will capture a “state of the art” summary of what is known about effective policies on health workforce distribution and retention, including from recent work by WHO and OECD, as well as current work underway funded by the ÉU. It will also draw from the recent themed series in “Human Resources for Health”.

The presentation will be a global overview (both high income and lower/middle income countries) presenting a synthesis of evidence, highlighting key policy interventions, and framed by existing typologies. Critical continuing gaps in the evidence base will also be highlighted.

